# Nursing competence in municipal in-patient acute care in Norway: a cross-sectional study

**DOI:** 10.1186/s12912-020-00463-5

**Published:** 2020-07-22

**Authors:** Torunn Kitty Vatnøy, Marianne Sundlisæter Skinner, Tor-Ivar Karlsen, Bjørg Dale

**Affiliations:** 1 Centre for Care Research, Southern Norway and Department of Health and Nursing Science, University of Agder, Box 509, NO-4898 Grimstad, Norway; 2 Center for Care Research, Eastern Norway and Department of Health Sciences NTNU – Norwegian University of Science and Technology, Box 191, NO-2802 Gjøvik, Norway; 3 Department of Health and Nursing Science, University of Agder, Box 509, NO-4898 Grimstad, Norway

**Keywords:** Acute care, Advanced practice nursing, Municipal health services, Older adults, Primary health care, Survey

## Abstract

**Background:**

The primary health care services are becoming increasingly complex, which presents challenges for the municipal nursing services. In Norway, municipal in-patient acute care (MipAC) has been introduced in all municipalities, and the competence at the services has been questioned. Few studies have examined the nursing services in the units. This study aims to get an overview of the nursing competence in those units across geographical regions, and different groups of organisation and localisation.

**Methods:**

A cross-sectional study was conducted, and an ad hoc questionnaire was distributed to first-line leaders in all the MipAC units in Norway. Data were collected in the period between 6 March 2019 to 6 June 2019. Measures to get an overview of the nursing competence were ratio of registered nurses (RNs) in staff, count of shifts with only one RN on duty and count of RNs with master’s degrees/specialisation. Descriptive comparative statistics were used.

**Results:**

Of all 226 first-line leaders invited to participate, 207 (91.6%) responded to the questionnaire. Overall a considerable variance across the sample was revealed. The median ratio of RNs in staff was 56 (IQR = 40–70), the count of shifts with only one RN on duty median 28 (IQR = 5–49), and the count of RNs with a master’s degree or specialisation median 3 (IQR = 0–5). The regions of Northern and Central Norway, MipACs located in nursing home and MipACs organised at long-term care units, showed significantly lower nursing competence in staff compared to the remaining institution and organisations.

**Conclusion:**

This study generates knowledge that can inform planning, priorities and interventions that may be initiated at all organisational and political levels concerning the MipAC services. An overall conclusion is that advanced nursing competence is lacking. The study also highlights the most urgent direction for improvements regarding nursing competence in the services. It seemed to be MipACs in Northern and Central Norway, and those located at nursing homes organised together with long-term care units, that needed improvements the most.

## Background

The general policy imperative to ensure accessible, attainable and safe health and care services to the population challenges health authorities’ planning, prioritisation and decision-making worldwide [1–3]. For positive patient outcomes and equitable access to high quality care to be achieved, a professional nursing workforce prepared to provide appropriate care is crucial [4–8]. According to the literature, patient-centeredness, clinical expertise, evidence-based practice, equity, expedient resource use [9] and ethical and respectful individualised holistic care characterise appropriate and quality care in nursing [10].

The estimates of an increasing aging population and accordingly increased incidence of diseases [2] motivate governments to search for solutions which use resources effectively and efficiently. One initiative in several European Union (EU) countries has been to implement health care reforms aimed at reducing the use of expensive hospital beds and strengthening primary health care services [1]. Hence, many services that previously were offered by the hospital have now been transferred to the municipalities. Consequently, the implementation of health care reforms has led to an increasingly complex and challenging arena for nursing care in the municipal health care services [4, 11]. The existing and estimated future shortage of registered nurses (RNs), in particular RNs with advanced nursing competence [6, 12, 13], represents some serious challenges.

In Norway, a key initiative in the 2012 Coordination Reform [14], was to establish Municipal in-patient Acute Care (MipAC) units in the primary health care services, in order to reduce the use of acute hospital services [15, 16]. Thus, from January 1, 2016, all Norwegian municipalities have been required by law to provide MipAC services to residents [17]. The typical patients admitted to these units were expected to be older patients with exacerbation of chronic diseases and infections [18]. By 2018, a total of 723 MipAC beds were registered in different units across the country. In total, 40,817 patients were admitted to MipACs in 2018; 69% were aged 67 or above, and 46% were the aged 80 and above. About 70% of the patients were admitted outside regular daytime hours [19, 20]. Although more efficient use of resources was put forward as an important goal, the national health authorities affirmed that the quality of the services should not be compromised. It was emphasised at the outset that the MipAC services should provide equal or even better healthcare to patients than hospital admission. Guidelines set down by the Directorate of Health [18] specified that patients who were treated in MipAC should not require advanced medical treatment. On the other side, it was stated that the services should provide both the competence and medical equipment required to handle patients with symptoms that may represent serious illness. This might, to some extent, represent some contradictions.

Apart from a requirement that RNs are present at all shifts, there are no minimum requirements for the nursing services in the MipACs [18].

Nursing competence is by nature an abstract phenomenon, and an agreed-upon definition might be difficult to attain [21]. However, the International Council of Nurses defines competence as: the “ongoing ability of a nurse to integrate and apply the knowledge, skills, judgment, and personal attributes required to practice safely and ethically in a designated role and setting” ([22], p.2). Nursing competence can be understood as an interaction between the individual nurse’s attributes and behaviour, i.e. what the nurse is and what she or he does [8, 23]. However, according to Sandberg & Pinnington [8] “competence is primarily socially rather than individually constituted” (p.1146). Although professional competence is personal in the sense that it is the person who enacts the competence that also embodies it, competence creates, exists, and evolves in a social-personal unity [8]. Research emphasises that there is a positive association between nursing competence and education [5, 21]. Vatnøy et al. [11] explored critical aspects of nursing competence in the context of the MipAC, and they found that in addition to the individual nurse’s attributes, nursing competence is also dependent on environmental and systemic factors in which a sufficiently qualified nursing staff is crucial [11]. The link between staffing level, i.e. the amount and composition of staff, and nursing competence has been clearly demonstrated in earlier studies [4, 5, 21, 24]. High nursing competence in staff has been shown to be associated with patient safety, quality of care, critical thinking, commitment, empowerment and positive practice environments.

In Europe, the understanding of the term professional nurse is a person who holds minimum a bachelor’s degree in nursing [5] and who is commonly referred to as a registered nurse (RN). Advanced nursing competence is described as anchored in advanced clinical skills, leadership, education and research in which their practice is characterised by capabilities including a combination of knowledge, values, skills and self-esteem which enable the person to handle change [25, 26]. The International Council of Nurses’ definition of advanced practicing nurse is “a registered nurse who has acquired the expert knowledge base, complex decision-making skills and clinical competencies for expanded practice, the characteristics of which are shaped by the context and/or country in which s/he is credentialed to practice. A master’s degree is recommended for entry level” [27] Advanced nursing competence is found to improve effectiveness, patient safety and quality in care [4, 24]. Advanced nursing competence in the context of MipAC is not defined although competence within emergency, geriatric, and mental health/psychiatric nursing is suggested as crucial [11, 18]. In addition, is it stated that the role of advanced practice nursing in primary health care comprises medical competence at a level beyond bachelor’s degree in nursing [4]. Further, studies clearly underpin the importance of a formally educated and qualified nursing workforce [5, 28–30] and warn against health care policies which undermine and neglect the need for a professional nursing workforce due to budget constraints and lack of RNs [5, 31].

Although admission rates have increased slightly over time, it is estimated that about 60% of the capacity in the Norwegian MipAC units remained unused in 2018 [15, 16, 19, 32]. A lack of confidence that the care providers in the units have sufficient competence has been put forward as a possible explanation for failing admission rates since the MipACs were introduced from 2012 onwards [16, 33]. According to estimates from Statistics Norway, the current shortage of nurses will continue to increase in the future [13]. A study has found that RNs constitute about one-third of the staff providing care for older people in Norwegian municipalities, while the remaining two-thirds consist of nurse assistants (NA) qualified through a degree from upper secondary school and assistants without formal health care training [12]. Therefore, a reasonable concern might be that the level of nursing competence among staff in the MipAC services is unsatisfactory, although we have limited knowledge of this [34, 35]. Although research shows a significant reduction in acute hospital admissions for older patients after the introduction of the MipAC beds [15, 16], the capacity appears to be underused. The degree of unused capacity appears to be related to localisation, organisation and geographical context [15, 16, 19]. Therefore, knowledge about how nursing competence in MipACs varies nationwide is important for strategic planning and making appropriate policy priorities and decisions.

## Methods

### Aim

The aim of this study was to get an overview of the nursing competence in Norwegian municipal in-patient acute care units across geographical regions, and different types of organisation and location.

### Research design

A cross-sectional study was conducted, and a web-based questionnaire was used for data collection.

### Setting

According to the legal requirements and recommendations provided by the Norwegian Directorate of Health [18], municipalities were free to organise MipAC services as appropriate to local contexts, such as population size, available resources and infrastructure. Consequently, the MipAC beds are organised in different ways and in different institutional locations [19, 36]. According to the Norwegian Directorate of Health [19], the MipAC units can be classified into four different categories of institutional locations; nursing homes, out-of-hours medical services (OMS), health-houses/local medical centre (HH/ LMC) and “Others”. The Norwegian Directorate of Health [19] reported that by the end of 2018, 137 MipAC services were located at nursing homes, 17 MipAC services were located at OMS, 29 MipAC services were located at HH/ LMCs and 26 MipAC were located at “Others”. In addition, the services in the different institutional locations might be organised together with long-term or short-term units. It is a statutory right in Norway to be assigned for an institutional long-term stay when needed, and the residents in long-term units at nursing homes are mainly frail older people in the age 80 or above [37]. Short-term stays might typically be in the transition between hospital and home and/or to assess the need for further care. OMS is a statutory primary health care service providing around the clock acute medical care to the citizens. Health-houses (HH) were established as a cooperation between two or more municipalities, with the rationale to bring together health services in a separate building to create more comprehensive and robust professional environments and to reduce operating costs. A local medical centre (LMC) is a service where one or more municipalities cooperate with the hospitals on the services. The category “Others” includes institutions which do not fall into the other categories, mainly short-term care units, e.g. intermediary units, and community hospital units [19, 20]. The four institutional categories (i.e. nursing homes, OMS, HH/ LMC, and “Others”) were based on the Directorate of Health’s classification of MipAC locations, as previously described [20]. Categories of geographical regions were extracted from Statistics Norway [38] according to the government’s classification of four regional health authorities: Northern Norway (citizens: 480000, area: 112000 km^2^), Central Norway (citizens: 700000, area: 56300 km^2^), Western Norway (citizens: 1100000, areal: 43432 km^2^) and South-Eastern Norway (citizens: 2900000, areal: 110000km^2^).

Approximately 59% of the units which serve patients admitted to MipACs are nursing homes and hold 28% of the MipAC capacity in Norway. HH/LMC and OMS make up 43% of the MipAC capacity and the remaining 20% are within the category “Others” [19]. In order to save costs, about 70% of the municipalities have chosen to offer MipAC in intermunicipal cooperation [19]. Regarding contracted hours per week for physicians, their presence or availability in the units has proven to vary widely [11, 15, 16].

### Data collection

The questionnaire was based on a previous qualitative study which explored critical aspects of nursing competence to care for older patients in MipACs in Norway [11]. That study revealed the importance of professional nurses in the staff, holding advanced competence, particularly within specialisation in emergency, geriatric and psychiatric nursing in these units. Furthermore, the study revealed that sufficient professional nurses in staff on duty enabling collegial support, opportunities for second opinions in patient assessment and control, e.g. when administering medication, was perceived as crucial for care quality and patient safety [11]. The questionnaire contained five items to identify characteristics of the MipAC units included in the study and ten items to give an overview of nursing competence. Table 1 presents an overview of the questions and measures included in the questionnaire.

**Table 1:** Items included in the questionnaire

**Sample characteristics questions**	**Measure/options**
Is the MipAC unit established as an intermunicipal cooperation?	Yes / No
How many of the total number of beds in your unit are defined as MipAC beds?	Count
What units are you leader of?^a^	a) Separate MipAC unit b) Short-term care unit c) Long- term care unit d) Both short-term and long-term units^b^
Are you the leader of more/other units? If so, which?	(open-ended)
How many hours per week is a physician contracted to be present in the MipAC?	Hours pr. week
**Nursing competence questions**	**Measure/options**
How many registered nurses (RNs) are responsible for the patients in the MipAC?	Count
How many nurse assistants (NAs) perform care for patients in the MipAC (i.e. assisting with meals, toilet visits, general care, observation and/or follow-up)	Count
How many assistants without formal health care training, perform care for patients in the MipAC (i.e. assisting with meals, toilet visits, general care, observation and/or follow-up)	Count
How many RNs have a master’s degree or specialisation in a) Emergency care nursing b) Geriatric nursing c) Mental health/psychiatric nursing d) other	Count
How many of the RNs have a master’s degree or specialisation in total = a + b + c + d.	Count
How many of the RNs have less than one year of clinical experience?	Count
Can you specify approximately how many a) day shifts b) evening shifts and c) night shifts were carried out with only one nurse on duty in the last four weeks? (despite presence of other staff with lower/no formal competence)	Count
How often does it happen that there is no nurse on duty in the MipAC?	Never/ Few times a year/ Monthly/ Weekly
When RNs call in sick or are absent, are their shifts in the MipAC covered by RNs?	Always/ Mostly/ Sometimes/Seldom/Never
Is there a written standard for minimum competence requirements for the nursing service in your MipAC?	Yes/ No

^a^ This question was included to get an overview of the type of organisations

^b^ Included in the category long-term care unit

The web-based software program SurveyXact™ was used for data collection.

### Sample

The sample included in this study was first line leaders in Norwegian MipAC units. First-line leaders, i.e. the operating leaders who were closest to the staff and who were responsible for the care provision in the 226 MipAC units, were invited to participate in the study. First line leaders were chosen as respondents as we assumed that they had the most accurate knowledge of the data we needed to investigate the research questions. If first-line leaders were absent for some reason, deputy leaders were asked to respond to the questionnaire. An e-mail list delivered by the Norwegian Directorate of Health provided information about the location of the MipACs. Contact information to first-line leaders were achieved through contacting the respective host municipalities. Potential respondents were contacted by telephone, provided with brief information about the study and asked to participate. Subsequently, we requested their e-mail addresses for distribution of the questionnaire. The questionnaires were distributed on 6 March 2019, and those who did not complete the questionnaire after the first e-mail were contacted by telephone to ensure receipt of the questionnaire. One e-mail reminder was subsequently sent out, and the survey was closed on 6 June 2019.

### Statistical analyses

The numeric variables are presented as median (Md) and interquartile range (IQR, 25–75), due to skewness in data distribution. Categorical data are presented as counts and percentages.

As the data were not normally distributed, we used two independent non-parametric samples, i.e. the Mann-Whitney U test and Kruskal-walls test to assess possible differences between groups. The resulting *p*-values were Bonferroni-corrected to avoid type 1 error [39]. Effect size was calculated for pairwise comparisons of the groups, using the z-statistics from the Mann-Whitney U test, as described in Fritz, Morris & Richler [40]. A large effect is proposed to be *r* ≥ 0.5, a medium effect *r* = 0.3–0.5, and a small effect *r* ≥ 0.1 [40]. The level of significance was set at *p* = 0.05. All tests were two-sided. Analyses were performed with the use of SPSS IBM statistics version 25.

### Ethical considerations

This study was carried out in accordance with the Declaration of Helsinki [41]. It was approved by the Norwegian Centre for Research Data on January 28, 2019 (ref. 815,471), and by the Faculty of Health and Sports Sciences at the University of Agder. Written information about the study, including the participants’ legal rights regarding participation and confidentiality, was provided. Participants were assured that it was voluntary to participate in the study and that they were free to withdraw from the study at any time. Completing and returning the questionnaire was considered as consent for participation.

## Results

**Fig. 1:**
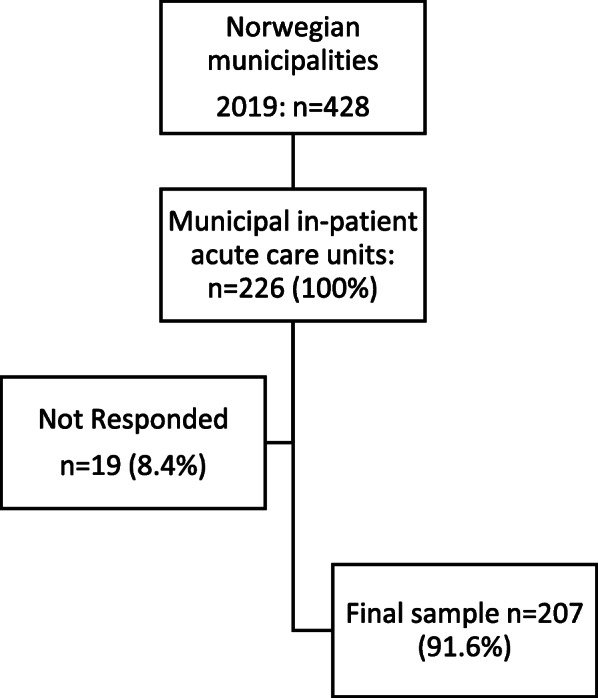
Flow chart including municipalities and recruitment of the first line leaders that constituted the sample

Figure 1 gives an overview of the MipAC population the non-responded and incomplete questionnaires, and the included sample. The final response rate was 91.6%.

### Characteristics of the MipACs

An overview of the institutional locations and geographical regions of the MipACs is presented in Table 2. In general, the analysis showed that the MipACs varied a lot regarding their institutional organisation. One third was hosted as intermunicipal cooperation. The MipACs were, generally, small, and almost half of the sample had less than two beds. About half of the MipACs were organised together with long-term care units. The median of the physicians contracted to be present in the units was 10 h per week. Nursing homes made up 68% of the MipAC location, and the majority of those were organised together with long-term care units.

**Table 2:** Characteristic of the MipACs across institutional locations and geographical regions

	Institutional localisations				Geographical regions				
	Nursing homes (*n* = 141)	OMS (*n* = 16)	HH/LMC (*n* = 26)	Others (*n* = 24)	Northern Norway (*n* = 56)	Central Norway (*n* = 42)	Western Norway (*n* = 32)	South- Eastern Norway (*n* = 77)	All regions (*n* = 207)
MipACs characteristics	*n (%)*	*n (%)*	*n (%)*	*n (%)*	*n (%)*	*n (%)*	*n (%)*	*n (%)*	*n (%)*
*MipAC whit intermunicipal cooperation (n = 207)*	26 (18.4)	11 (64.7)	18 (69.2)	12 (50)	10 (17.9)	14 (33.3)	10 (31.3)	33 (42.9)	67 (32.4)
*Two or more MipAC beds (n = 205)*	55 (39)	15 (88.2)	25 (96.2)	18 (75)	22 (40)	15 (36.6)	18 (56.3)	58 (75.3)	113 (55.1)
*Organisation (n = 205)*									
Separate MipAC unit	2 (1.4)	5 (31.3)	5 (19.2)	4 (16.7)	1 (1.8)	3 (7.1)	3 (9.4)	9 (11.7)	16 (7.8)
Short-term care units	43 (30.9)	11 (68.8)	20 (76.9)	15 (62.5)	20 (33.9)	10 (23.8)	18 (56.3)	42 (54.5)	89 (43.4)
Long- term care units	94 (67.6)	0 (0)	1 (3.8)	5 (20.8)	35 (64.3)	28 (66.7)	11 (34.4)	25 (32.5)	101 (48.8)
	*Md (IQR)*	*Md (IQR)*	*Md (IQR)*	*Md (IQR)*	*Md (IQR)*	*Md (IQR)*	*Md (IQR)*	*Md (IQR)*	*Md (IQR)*
*Counts of MipAC beds*	1 (1–2) (*n* = 139)	5.5 (4–15.2)	5 (3.7–9.2)	2 (1.2–4.7)	2 (1–2)	2 (1–2.5)	2 (1–4)	3 (1.8–5)	2 (1–4)
*Physician contracted to be present, hours per week (n = 203)*	7.0 (3–19) (*n* = 138)	33.7 (10–165)	37.5 (22.7–45.6)	21.0 (1–52) *n* = 23	6.5 (0.5–20.5)	7.2 (2.3–33.8)	18 (5–37.5)	16.4 (6–40)	10 (4–35.5)

Northern and Central Norway represented the regions with the fewest MipAC beds; with respectively 60 and 63% holding less than two MipAC beds. These regions had also the highest number of MipACs organised together with long-term care units, and the lowest median of the physicians contracted to be present in the units. Further, Northern Norway had the lowest number of MipACs with intermunicipal cooperation (18%).

### Nursing competence in MipAC

An overview of nursing competence across institutional categories and geographical regions is presented in Table 3. In total, the proportion of RNs in staff ranged between 9 to 100% in the units. Nursing homes showed the lowest ratio of RNs to other staff and had also the highest number of staff without formal health care training. A similar pattern was also found in the regions of Northern and Central Norway.

**Table 3:** Professional nursing competence across institutional locations and geographical regions

	Institutional locations				Geographical regions				
	Nursing homes (*n* = 141)	OMS (*n* = 16)	HH/LMC (*n* = 26)	Other locations (*n* = 24)	Northern Norway (*n* = 56)	Central Norway (*n* = 42)	Western Norway (*n* = 32)	South-Eastern Norway (*n* = 77)	Total (*n* = 207)
Nursing competence in MipAC	*Md (IQR)*	*Md (IQR)*	*Md (IQR)*	*Md (IQR)*	*Md (IQR)*	*Md (IQR)*	*Md (IQR)*	*Md (IQR)*	*Md (IQR)*
*Registered nurses (RNs)* (*n* = 203)	10 (8–14)	20 (15–35)	17.5 (12.7–22.3)	14 (11–19)	9 (7.3–11.8)	10.5 (7–15.7)	15 (5–50)	15 (10–20)	12 (8–17)
*Nurses assistants (NA*) *(n = 200)*	9 (5–12)	1.5 (0–6)	4.5 (1.8–7.3)	4 (0–6)	7 (4–12)	8 (4–11)	6 (2–8.5)	7 (3–10)	7 (3–10.8)
*Staff without formal health care training (n = 200)*	2 (0–5)	0 (0–0)	0 (0–1.3)	0 (0–2)	2 (0–5)	3 (0–6)	0 (0–11)	0 (0–3)	1 (0–4)
*Proportion of RNs in staff (n = 203)*	50 (35–63.6)	92.2 (76.3–100)	74.5 (58.5–91)	75 (56.2–100)	47.4 (34.4–66.6)	47.2 (33.7–60.8)	64.7 (55.3–85.7)	65.7 (49–86.2)	56 (40–78)
*RNs with less than one years’ experience* (*n* = 200)	0 (0–1)	0 (1–2.75)	0 (0–1)	1 (0–1)	0 (0–1)	0 (0–0)	0 (0–1)	0 (0–1)	0 (0–1)
*RNs with additional* education (*n* = 203)									
Emergency nursing	0 (0–1)	1 (0–1.75)	0 (0–1)	0 (0–2)	0 (0–1)	0 (0–1)	0 (0–1)	0 (0–1)	0 (0–1)
Geriatric nursing	0 (0–1)	0 (0–1)	0 (0–1)	0 (0–1)	0 (0–1)	0 (0–1)	0 (0–1)	0 (0–1)	0 (0–1)
Mental health/psychiatric nursing	0 (0–1)	1 (0–1.75)	0 (0–1)	0 (0–1)	0 (0–1)	0 (0–1)	0 (0–1)	0 (0–1)	0 (0–1)
*Total additional educated RNs (n = 199)*	3 (2–4)	3 (5–7)	3 (1–5.5)	4 (2–5)	3 (0–9)	4 (2–5)	4 (1–5)	3 (1–3)	3 (0–5)
*Work shifts with one RN on duty over the last four weeks (n = 200)*									
Daytime	5 (0–9)	0 (0–1.75)	0 (0–0.25)	0 (0–8)	8 (0–10)	4 (0–8)	0 (0–4.3)	0 (0–7)	3 (0–8)
Evening time	10 (2–20)	0 (0–1.75)	0 (0–3.3)	1 (0–10)	12 (2–20)	10 (2–17)	2 (0–5)	1 (0–10)	5 (0–15.8)
Nighttime	28 (5–28)	0 (0–5.5)	0 (0–28)	14 (0–28)	28 (15–28)	17.5 (0–28)	3.5 (0–28)	12 (0–28)	20 (0–28)
*Work shifts in total with one RN on duty over the last four weeks*	34 (15–54)	0.5 (0–10)	1.5 (0–28)	16 (0–36)	43 (25–58)	33 (2.7–52.8)	12.5 (0.8–30.3)	15 (0–42)	28 (5.3–49)
	*n (%)*	*n (%)*	*n (%)*	*n (%)*	*n (%)*	*n (%)*	*n (%)*	*n (%)*	*n (%)*
*Nursing competence standard requirements (n = 193)*	68 (50.4)	13 (81.3)	16 (61.5)	11 (47.8)	18 (35.3)	20 (51.3)	20 (66.7)	50 (68.5)	108 (56)
*Shift without RNs on duty (n = 200)*									
- Never	95 (70.4)	14 (87.5)	25 (96.2)	18 (78.3)	39 (70.9)	25 (59.5)	26 (86.7)	62 (82.7)	152 (76)
- A few times a year	24 (17.8)	2 (12.5)	1 (3.8)	2 (8.7)	9 (16.4)	8 (20)	3 (10)	9 (12)	29 (14.5)
- Monthly	10 (7.4)	0 (0)	0 (0)	2 (8.7)	4 (7.3)	5 (12.5)	1 (3.3)	2 (2.7)	12 (6)
- Weekly	6 (4.4)	0 (0)	0 (0)	1 (4.3)	3 (5.5)	2 (4.8)	0 (0)	2 (2.7)	7 (3.5)
*Vacant RNs shift (due to sick leave,* etc.*) covered by RNs (n = 200).*									
- Always	65 (48.1)	12 (75)	9 (34.6)	11 (47.8)	28 (50.9)	17 (42.5)	14 (46.7)	38 (50.7)	97 (48.5)
- Mostly	42 (31.1)	3 (18.8)	15 (57.7)	8 (34.8)	20 (36.4)	14 (35)	11 (36.7)	23 (30.7)	68 (34)
- Sometimes	19 (14.1)	1 (6.3)	2 (7.7)	2 (8.7)	5 (8.9)	6 (15)	4 (13.3)	9 (12)	24 (12)
- Rarely	7 (5.2)	0 (0)	0	1 (4.3)	2 (3.6)	3 (7.5)	1 (3.3)	3 (4)	9 (4.5)
- Never	1 (0.7)	0	0	1 (4.3)	0	0	0	2 (2.7)	2 (1)

The number of RNs holding a master’s degree or a nursing specialisation, was in general low across institutional locations and geographical regions, and the median score was 0 within emergency, geriatric and mental health/psychiatric nursing specialisations.

The median for shifts with only one RN on duty the last 4 weeks was Md = 28 for the total sample, and highest for night shifts with Md = 20. Nursing homes and Northern Norway had the highest medians, with Md = 34 and Md = 43 respectively on all shifts in total, and Md = 28 and Md = 28 respectively on night shifts.

Seven MipACs reported that they weekly had shifts with no RNs on duty, six of which were nursing homes. Of the total sample, 24% reported not having RNs on all shifts. Further, the majority of the respondents reported that vacant RN shifts (for example due to absence or sick leave) were always or mostly covered by RNs, while 18% reported that this was the case sometimes, seldom or never. Nursing homes most frequently reported that vacant RN shift sometimes, seldom or never were covered by RNs. Furthermore, 56% of the total sample reported that their unit did have minimum nursing competence standard requirements for the MipAC.

### Differences in nursing competence between groups

The ratio of RNs to other staff in nursing homes was significantly lower compared to the remaining institutional locations. Regarding geographical regions, we found a significantly lower ratio of RNs in Northern and Central Norway compared to South-Eastern and Western Norway. When comparing the three categories of organisation, we found significant lower ratio of RNs to other staff in short-term care units (Md = 67) compared to separate MipACs units (Md = 91), although the difference was small. Long-term care units had the lowest ratio of RNs to other staff (Md = 44) compared to short-term and separate MipACs units. The differences were medium and large, respectively.

Nursing homes had significantly more shifts with only one RN on duty compared to the remaining institutional locations (Md values are presented in Table 3). Further, Table 3 shows that the MipACs organised together with long-term care units (Md = 43) had more shifts with only one RN on duty compared to short-term (Md = 12) and separate MipAC units (Md = 0). MipACs organised as intermunicipal cooperation showed significant higher ratio of RNs to other staff and lower number of shifts with only one RN on duty than those not organised as intermunicipal cooperation. This was also the case for MipACs who had two MipAC beds or more (Table 4).

**Table 4:** Differences in nursing competence between groups

	Ratio of RNs in staff		Shifts with one RN on duty		Nightshifts with one RN on duty		Dayshifts with one RN on duty	
Comparation between groups	r	Adj. *p* value	r	Adj. p value	r	Adj. p value	r	Adj. p value
**Institutional locations**								
Nursing homes- Others	0.31	**< 0.001**	0.22	**0.028**	0.19	0.102		na
Nursing homes- HH/LMCs	0.39	**< 0.001**	0.37	**< 0.001**	0.29	**0.001**		na
Nursing homes- OMS	0.43	**< 0.001**	0.33	**< 0.001**	0.31	**< 0.001**		na
Others- HH/LMCs	0.09	1.0	0.19	1.0	0.13	1.00		na
Others- OMS	0.26	1.0	0.22	1.0	0.24	1.00		na
HH/LMCs- OMS	0.17	1.0	0.03	1.0	0.11	1.00		na
**Organisation categories**								
Short-term care units -Separate MipAC units	0.27	**0.017**	0.21	0.094	0.14	0.5		na
Long-term care units -Short-term care unites	0.43	**< 0.001**	0.44	**< 0.001**	0.31	**< 0.001**		na
Long-term care units -Separate MipAC units	0.56	**< 0.001**	0.52	**< 0.001**	0.37	**< 0.001**		na
**Geographical region**								
Central Norway-Southern-Eastern Norway	0.26	**0.022**		na		na		na
Central Norway-Western Norway	0.34	**0.020**		na		na		na
Northern Norway -Southern-Eastern Norway	0.26	**0.017**		na		na		na
Northern Norway-Western Norway	0.31	**0.019**		na		na		na
Central Norway-Northern Norway	0.02	1.000		na		na		na
Southern-Eastern Norway-Western-Norway	0.06	1.000		na		na		na
	r	p-value	r	p-value	r	p-value	r	p-value
Not intermunicipal MipAC- Intermunicipal MipAC	**0.45**	**< 0.001**		na	0.32	**< 0.001**	0.20	**0.005**
Less than two MipAC beds or more	0.51	**< 0.001**	0.34	**< 0.001**		na	0.28	**< 0.001**

Not applicable = na, due to heterogeneity of variance as described in Field (2009). Comparing evening shifts between the group categories was not applicable. A large effect is proposed to be *r* ≥ 0.5, a medium effect *r* = 0.3–0.5, and a small effect *r* ≥ 0.1 (Fritz et al. 2012)

## Discussion

The aim of this study was to get an overview of the nursing competence in Norwegian municipal in-patient acute care units across geographical regions and different types of organisation and location. The overall professional nursing competence in the MipAC services depends on a sufficiently qualified nursing staff [11] prepared to address an increasingly complex and challenging arena for nursing care [4, 11]. Along with the individual nurse’s attributes and behaviour [23], nursing competence is situationally and socially related [8]. Competence is associated with educational level [5, 21, 24] and can be characterised as high or low, sufficient or insufficient [8]. This study investigates the number and presence of RNs in staff throughout day, evening and night shifts as well as the amount of RNs holding a formal competence beyond bachelor’s degree, i.e. a master’s degree or a nursing specialisation.

The number of RNs at the MipACs holding master’s degree or specialised competence was in general low. The lowest ratio of RNs to other staff and the highest frequency of shifts with only one RN on duty was found in MipACs located at nursing homes, organised together with a long-term care unit, and in MipACs in the Northern and Central regions of Norway. The results are in line with literature which reports a lack of RNs [13] and of RNs holding advanced nursing competence in primary health care services [4, 12].

The low ratio of RNs to other staff found in our study, combined with increased responsibilities and complexity in nursing practice in the MipACs, may affect the recruitment of RNs and consequently the quality of nursing service in the units. The many benefits of a high proportion of RNs working bedside in general is described in several studies [5, 42]. A high ratio of RNs to other staff have showed positive impact on patient safety and patient satisfaction, but also on nurses’ job satisfaction and prevention of burnout as well as expensive and disruptive turnover of staff [5, 42]. Moreover, the results indicate that there is considerable time when there is only one RN on duty. It is particularly worrying that one RN on duty were most frequent outside ordinary daytime (e.g. evenings and nights), which is the time when 70% of the patients are reported to be admitted [20]. In accordance with previous research [11, 15, 16], we also found considerable variations in physicians contracted to be present in MipACs, which indicates that assessments and critical decisions often may depend on the RNs in the units. This underlines the great responsibility that lies with the RNs, and which might represent a burden for RNs working alone on duty. Along with the individual RNs’ level of expertise, the overall nursing competence and quality of care depend on sufficient nursing staff in a professional and collaborating atmosphere, acknowledged and supported by a professional leadership [8, 11, 43] The benefits of having collegial support to handle the workload is described in several studies [5, 42, 44]. This includes, among other things, to assess and discuss patients and different situations in order to make accurate decisions and interventions, as well as access to qualified second opinions [5, 42, 44].

The lack of RNs holding master’s degrees or specialised competence, as revealed in our study, may be viewed as worrying because nurses’ responsibilities and complexity in primary health care services have increased [4]. Advanced nursing competence is recommended to safeguard patients in complex and advanced settings in [11, 26, 28]. Because older people in a condition characterised by disability, frailty and comorbidity [45] are the main patient group admitted to the MipACs [2, 11, 19], nurses holding competence and capabilities to observe, assess, make decisions and handle acute care situations, as well as general professional care, are required [11, 46]. Nurses holding advanced nursing competence is also shown to safeguard patients with complex health care needs in rural areas, as they are found to deliver high adherence to clinical guidelines and provide diagnostic accuracy [24]. One of the main goals with the MipACs was to provide equal or better healthcare services to patients where they live, compared to services provided in hospitals. A significant portion of the population in Norway lives in rural areas and many of the MipACs serve this population. Therefore, it is important that the expertise is present. Our study showed that being organised in an intermunicipal cooperation, or in a MipAC with two or more beds, are advantageous with regard to the ratio of RNs to other staff as well as having more than one RN on duty. Nonetheless, our study also showed that 68% of the MipACs in the sample were not organised in cooperation with other municipalities. A rationale behind this might be the costs for ambulance transport due to large distances, especially in the Northern and Central Norway. In addition, costs related to purchasing the care services from a host municipality are perceived to balance out some of the economic benefits of co-locating services [47]. Besides, many municipalities might have concluded that it would be in their patients’ best interest to be treated as close as possible to own homes. This is in line with the study of Leonardsen et al. [48], who found that patients experienced geographical proximity, a pleasurable atmosphere and time allocated for care as quality criteria in the MipACs. These aspects might highlight the necessity to upscale the professional nursing staff in order to assure care quality in the MipACs in small and rural units. According to our study, this is particularly critical in the Northern and Central regions of Norway. Although the lack of nursing competence revealed in our study does not necessarily provide evidence for compromised safety and quality in patient care in Norwegian MipACs, it might support the assumptions that low competence can partly explain the low use of MipAC beds in general [15, 16, 33]. Considering the low utilisation of the MipAC capacity, older people in an acute condition are still, to a large extent, referred to hospital emergency departments. MipAC services might be an advantageous alternative to hospital admission for the older adults, given that the services are sufficiently staffed with competent professionals providing individualised holistic quality care to the patients. Studies show that hospital emergency departments often fail to provide quality care for geriatric patients [49–51] because they are not well organised to meet older people’s needs [49]. Older patients’ conditions are likely to be regarded as less critical, they are often referred to wait for treatment [50], and adverse events in emergency departments are more frequent in this group of patients [51]. Previous research indicates that nurses working in geriatric acute care units show more favourable attitudes to older people, holding more gerontological knowledge compared to nurses working at general acute medical units [52]. Furthermore, RNs working in municipal health care services are found to focus more on the patients’ life situation in a holistic perspective compared to those working in hospital emergency departments [53].

Our results indicate that MipACs organised together with short-term care units hold a higher level of nursing competence compared to those organised together with long-term care units. Thus, the organisational structure seemed to be important. Our results also showed that the incidence of night shifts with only one RN on duty indicates potential for improvements. One explanation to the high amount of nightshifts with only one RN, might be that requirements to professional nursing competence in the units have not been changed according to the increased complexity in the services. The culture, workload and leadership in the units are also found to be important for implementing internal changes and professional development [54]. It is, therefore, an important responsibility for the leaders of the MipACs to initiate, plan and facilitate for sufficient professional nursing staff in the units. However, to gain knowledge about how management and leadership impact professional nursing competence in MipACs, further research is needed.

### Limitations

First, it is important to underline the inherent limitations of a cross-sectional study design. Its contribution is only to describe the situation of interest and studying causal interferences and changes over time is not possible. Cross-sectional data represents an overview of a phenomenon or situation at one particular point in time, and the identical procedure conducted at another time could give different results. Furthermore, although the response rate in our survey was high, some analyses were unavailable due to heterogeneity of variance in the data [39].

Second, a complete overview of all MipAC locations was difficult to achieve. Although an updated list including the host municipalities provided by the Directorate of Health was used during the recruitment of participants, we experienced that the list was not consistent with how the municipalities were composed. Although considerable effort was made to identify and include the whole population, there might be units which serve MipACs that have been missed.

Third, this study examined nursing competence, which in nature is an abstract, multifaceted phenomenon, and definitory consensus is lacking [21]. There might be several other ways to study nursing competence in the context of the MipAC, both in terms of definitions and approaches. This study examines only a selection of variables, which nevertheless are assumed to be important aspects of nursing competence in the MipAC units.

## Conclusion

One consequence of the implementation of health reforms in Europe is an increasingly complex and challenging arena for nursing practice in primary health care. This study investigates, and gives an overview of, the professional nursing competence in the Norwegian MipACs in primary health care after the implementation of the Coordination Reform. The reform aimed to reduce use of expensive hospital beds and facilitate equitable access to safe and secure MipAC services close to the patient’s home. This study reveals significant differences in professional nursing competence in the MipAC services in Norway. Thus, it generates knowledge that can inform planning, priorities and interventions that may be initiated at all organisational and political levels concerning the MipAC services. An overall conclusion is that advanced nursing competence is lacking. The study also highlights the most urgent direction for improvements regarding nursing competence in the services. It seemed to be MipACs in Northern and Central Norway, and those located at nursing homes organised together with long-term care units, that needed improvements the most.

## Data Availability

The dataset and analyses are not currently publicly available as further articles based on the dataset are planned. However, the materials could be available from the corresponding author upon reasonable requests.

## Abbreviations

MipAC: Municipal in-patient acute care
RN: Registered nurse educated at the minimum level of bachelor’s degree
NA: Nurse assistant
OMS: Out-of-hours medical services
HH/LMC: Health house/local medical centre
IQR: Interquartile Range
EU: European Union
