# Correction to: Nursing competence in municipal in-patient acute care in Norway: a cross-sectional study

**DOI:** 10.1186/s12912-020-00519-6

**Published:** 2020-12-15

**Authors:** Torunn Kitty Vatnøy, Marianne Sundlisæter Skinner, Tor-Ivar Karlsen, Bjørg Dale

**Affiliations:** 1 Centre for Care Research, Southern Norway and Department of Health and Nursing Science, University of Agder, Box 509, NO-4898 Grimstad, Norway; 2 Center for Care Research, Eastern Norway and Department of Health Sciences, NTNU – Norwegian University of Science and Technology, Box 191, NO-2802 Gjøvik, Norway; 3 Department of Health and Nursing Science, University of Agder, Box 509, NO-4898 Grimstad, Norway

**Correction to: BMC Nurs 19, 70 (2020)**

**https://doi.org/10.1186/s12912-020-00463-5**

Following publication of the original article [1], the authors identified an error in author affiliation 2.

Current author affiliation 2:

Centre for Care Research, Southern Norway and Department of Health Sciences NTNU, Norwegian University of Science and Technology, Box 191, NO-2802, Gjøvik, Norway

Correct author affiliation 2:

Center for Care Research, Eastern Norway and Department of Health Sciences NTNU – Norwegian University of Science and Technology, Box 191 NO-2802 Gjøvik, Norway

The original article has been updated.
